# The short-term efficacy of high flow nasal oxygen therapy on cardiovascular surgical patients: a randomized crossover trial

**DOI:** 10.1186/s12871-022-01883-3

**Published:** 2022-10-29

**Authors:** Deguchi Shiho, Yusuke Kusaka, Shoko Nakano, Osamu Umegaki

**Affiliations:** Department of Anesthesiology, Osaka Medical and Pharmaceutical University, Daigaku-machi 2-7, Takatsuki, Osaka, 569-8686 Japan

**Keywords:** High flow nasal oxygen therapy, Venturi mask, Cardiovascular surgery, Oxygen therapy after extubation

## Abstract

**Background:**

Oxygen therapy after extubation in the intensive care unit (ICU) is essential in order to maintain adequate oxygenation, especially in patients who have undertaken cardiovascular surgery. A Venturi mask (VM) has been routinely used as an oxygen therapy in the ICU. Recently, however, the high flow nasal cannula (HFNC) has become available, and this device can deliver up to 60 L/min of humidified oxygen. The aim of this study is to evaluate the short-term efficacy between HFNC and VM in cardiovascular surgical patients.

**Methods:**

Forty patients who underwent cardiovascular surgery were randomized to either protocol A (HFNC followed by VM) or protocol B (VM followed by HFNC). After 60-minutes of use with either device, arterial blood gas analysis was performed, and the PaO_2_/FiO_2_ ratio (PFR) was calculated. Simultaneously, physiological data (respiratory rate, heart rate, mean arterial pressure, continuous cardiac index, and mixed venous oxygen saturation) were recorded. During this procedure, FiO_2_ and gas flow were maintained at a fixed rate. These variables were compared by using the paired t-test, and a *p* value < 0.05 was considered significant. All data were expressed as mean (standard deviation).

**Results:**

Thirty-five patients (17 from protocol A and 18 from protocol B) were enrolled, and 5 patients were excluded from analysis in accordance with the exit criteria. PaO_2_ was significantly higher in the HFNC group than in the VM group [101.7 (25.9) vs. 91.8 (23.0), mean difference 9.87 (18.5), 95% confidence interval 3.5 to 16.2, *p* = 0.003]. Moreover, PFR was significantly higher in the HFNC group than in the VM group [265.9 (81.4) vs. 238.7 (68.5), *p* = 0.002]. Moreover, PaCO_2_ was significantly lower in the HFNC group than in the VM group [33.8 (3.5) vs. 34.7 (2.9), *p* = 0.033]. The respiratory rate was significantly lower in the HFNC group than in the VM group [18 (4) vs. 21 (4), *p* = 0.006], and no significant differences were seen in any of the other parameters.

**Conclusions:**

Compared to VM, HFNC ameliorated oxygenation function and decreased patients’ effort in breathing. The hemodynamic state did not differ between HFNC and VM. Therefore, HFNC can be used safely in cardiovascular surgical patients.

**Trial registration:**

This trial was registered with the UMIN Clinical Trials Registry (ID UMIN000016572).

## Background

Oxygenation and gas exchange occasionally deteriorate after cardiovascular surgery due to the usage of cardiopulmonary bypass and perioperative blood transfusion. Postoperative optimal oxygen delivery in the intensive care unit (ICU) is essential for adequate oxygenation in order to prevent reintubation and postoperative adverse respiratory events. It is crucial to adjust the fraction of inspired oxygen (FiO_2_) and the oxygen flow rate when performing postoperative oxygen therapy. Minimalizing FiO_2_ is important in order to avoid absorption atelectasis – one of the possible respiratory complications after cardiovascular surgery [1]. In general, an oxygen flow rate of 30 L/min is necessary to accurately provide pre-specified FiO_2_ and to prevent the lungs from drawing ambient air. In recent years, the high flow nasal cannula (HFNC), which can deliver up to 60 L/min of humidified oxygen, has become available and widely used in the perioperative field. On the other hand, the Venturi mask (VM) has been routinely used as a high flow oxygen device for quite a while. VM needs a low flow rate of oxygen in order to create a large total flow rate, at predictable FiO_2_, entraining room air via the Venturi effect. Both devices can precisely regulate both FiO_2_ and flow rate and, therefore, both are thought to be comparable as a postoperative high-flow oxygen device. The aim of this study is to evaluate the short-term efficacy between HFNC and VM in cardiovascular surgical patients.

## Methods

This randomized crossover trial was performed in the ICU (8 beds) of Osaka Medical College. The protocol of this study was approved by the institutional ethics committee of Osaka Medical College (file number: RIN89–1635) and registered with the UMIN Clinical Trials Registry (ID UMIN000016572, February 18th, 2015). Written informed consent was obtained from each patient. From February to August 2015, the authors recruited 40 patients who underwent scheduled cardiovascular surgery using cardiopulmonary bypass with median sternotomy and mild hypothermia. After the operation, all participants were admitted to the ICU and received mechanical ventilation under a continuous infusion of sedatives (propofol and dexmedetomidine). The day after surgery, patients who fulfilled the following criteria before extubation were eligible for the randomization of this study: arterial blood pH 7.35 to 7.45, PaO_2_/FiO_2_ ratio (PFR) ≧ 250 (mmHg), FiO_2_ ≦ 0.4, positive end-expiratory pressure (PEEP) ≦ 5 cmH_2_O, and pressure support (PS) ≦ 5 cmH_2_O. Patients were excluded if they had bronchial asthma, chronic obstructive pulmonary disease, hemodynamic instability, end-stage renal failure requiring hemodialysis, or a duration of postoperative mechanical ventilation in the ICU > 24 hours. In this study, we used an Aerosol mask® (Smith Medical inc. Minnesota, US) and an EZ-Water® nebulizer (Japan Medicalnext Co., Ltd., Osaka, Japan) as a humidification and Venturi system. The HFNC system includes OA2060® (Sanyu technology Co., Ltd., Saitama, Japan) as an air/oxygen blender, and an F&P 850® system (Fisher & Paykel Healthcare, Co., Ltd., Auckland, New Zealand) as a circuit.

**Fig. 1:**
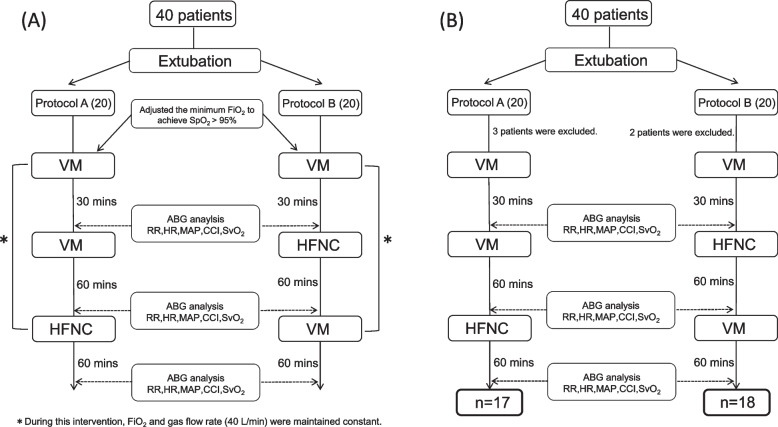
**A** Patient flowchart of this trial. VM, Venturi mask; HFNC, high flow nasal cannula; RR: respiratory rate; HR, heart rate; MAP, mean arterial pressure; CCI, continuous cardiac index; SvO_2_ mixed venous oxygen saturation. **B** Patient flow after randomization. VM, Venturi mask; HFNC, high flow nasal cannula; RR: respiratory rate; HR, heart rate; MAP, mean arterial pressure; CCI, continuous cardiac index; SvO_2_, mixed venous oxygen saturation

Before the study, we measured the oxygen flow rate of VM (Table 1), which is necessary to provide a total gas flow rate of 40 L/min using HALOSCALE® flowmeter (nSpire Health Ltd., Hertford, UK). After extubation, patients were provided with oxygen by VM at a rate of 40 L/min for 30 minutes. Targeted minimum FiO_2_ was adjusted to maintain SpO_2_ ≧ 95%, selecting from 0.33, 0.35, 0.4 and 0.5 (Table 1). After stabilization of this 30-minute oxygen administration, the arterial blood gas (ABG) analysis (pH, PaO_2_, PaCO_2_, and HCO_3_^−^) was performed, and PFR was calculated simultaneously. Respiratory rate (RR), heart rate (HR), mean arterial pressure (MAP), continuous cardiac index (CCI), and mixed venous oxygen saturation (SvO_2_) were also recorded. RR was measured using thoracic impedance pneumography (Life Scope®, Nihon Kohden, Tokyo, Japan). CCI and SvO_2_ were measured by a pulmonary artery catheter inserted after the induction of general anesthesia in the operating room. If the patients remained respiratorily and hemodynamically stable, they were then randomized into either protocol A (VM for 60 minutes, followed by HFNC for 60 minutes) or protocol B (HFNC for 60 minutes, followed by VM for 60 minutes) (Fig. 1A). During this intervention, FiO_2_ and a total gas flow rate of 40 L/min were maintained fixed and in similar fashion with the stabilization interval described above (Fig. 1A). At the end of the period of each oxygen device, PFR was calculated from the ABG analysis, and RR, HR, MAP, CCI, and SvO_2_ were recorded. The primary outcome of this study was PaO_2,_ and the secondary outcomes were PFR, PaCO_2_, RR and hemodynamic parameters.

**Table 1:** Necessary oxygen flow rate for VM to provide total gas flow of 40 L/min

FiO_2_	33%	35%	40%	50%
Flow rate (L/min)	6	7	10	15

Randomization was performed using sequentially numbered sealed envelopes to preserve allocation concealment. The number of patients in this trial was calculated as follows: the overall average trial PaO_2_ in this setting was 95 ± 20 mmHg and obtained from our preliminary data. Thirty-three subjects were needed to show a PaO_2_ difference of 10 mmHg at a significance level of 0.05 and a power of 80%. The sample size was inflated to 40 patients to account for withdrawals and loss. Data are described as mean (standard deviation) and numbers with proportions (%), where appropriate. Baseline data of each protocol was assessed by the Welch’s t and Chi-square tests. Outcome variables were compared using the paired t test. Statistical analyses were performed separately for protocol A and protocol B, considering that a carry-over effect affected the results of this study.

All tests were two-tailed, and a *p* value < 0.05 was considered to be statistically significant.

## Results

A total of 40 patients were recruited, and 20 each were randomized into either protocol A or B. Three patients from protocol A and two from protocol B were excluded from analyses due to early discharge from the ICU (Fig. 1B). Table 2 shows the patient background of each protocol at randomization, including age, gender, height, body weight, body mass index, ventilation time before extubation and type of operation. Table 3 shows the result of the ABG analysis and baseline physiologic data of each protocol before intervention.

**Table 2:** Patient background of each protocol at randomization

	Protocol A		Protocol B		*p* value
	mean	(SD)	mean	(SD)	
Age	66.0	(11.0)	71.9	(8.9)	0.100
Gender (male/female)	11/6		9/9		0.380
Height (cm)	162.2	(8.8)	157.0	(11.8)	0.156
Body weight (kg)	60.3	(12.1)	56.7	(14.6)	0.444
BMI (kg/m^2^)	21.3	(5.8)	22.8	(3.7)	0.401
Ventilation time (min)*	1057.8	(333.7)	983.2	(204.7)	0.436
Operation time (min)	368.4	(149.4)	384.4	(89.8)	0.708
Operation					0.187
CABG	4		8		
Valve	11		10		
Others	2		0		

*SD* Standard deviation, *BMI* Body mass index, *CABG* Coronary artery bypass grafting

**Table 3:** ABG analysis and baseline physiological data of each protocol before intervention

	Protocol A		Protocol B		*p* value
	mean	(SD)	mean	(SD)	
PaO_2_ (mmHg)	95.9	(23.4)	97.6	(25.7)	0.380
FiO_2_ (0.35/0.4/0.5)	9/7/1		9/4/5		0.177
PFR (mmHg)	252.8	(62.4)	230.0	(51.6)	0.248
PaCO_2_ (mmHg)	33.5	(3.4)	34.9	(2.9)	0.923
pH	7.428	(0.02)	7.434	(0.04)	0.641
HCO_3_^−^ (mEq/L)	22.4	(2.4)	23.1	(1.7)	0.606
RR (rates/min)	19.2	(4.2)	20.4	(4.7)	0.428
HR (beats/min)	92.4	(11.6)	94.6	(9.0)	0.073
MAP (mmHg)	65	(10)	66	(8)	0.940
CCI (L/min/m^2^)	3.8	(0.8)	3.5	(0.6)	0.199
SvO_2_ (%)	71.9	(10.1)	73.2	(4.5)	0.674

*ABG* Arterial blood gas, *SD* Standard deviation, *PFR* PaO_2_/FiO_2_ ratio, *RR* Respiratory rate, *HR* heart rate, *MAP* Mean arterial pressure, *CCI* Continuous cardiac index, *SvO*_*2*_ Mixed venous oxygen saturation

**Fig. 2:**
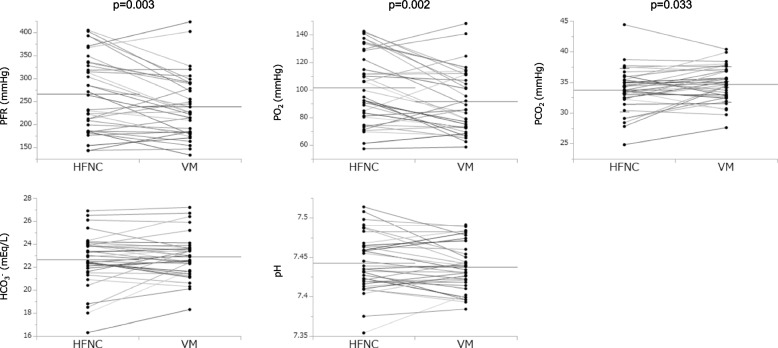
HFNC vs. VM (ABG analysis). HFNC, high flow nasal cannula; VM, Venturi mask; PFR, PaO_2_/FiO_2_ ratio. Horizontal lines indicate the mean value of each device

**Fig. 3:**
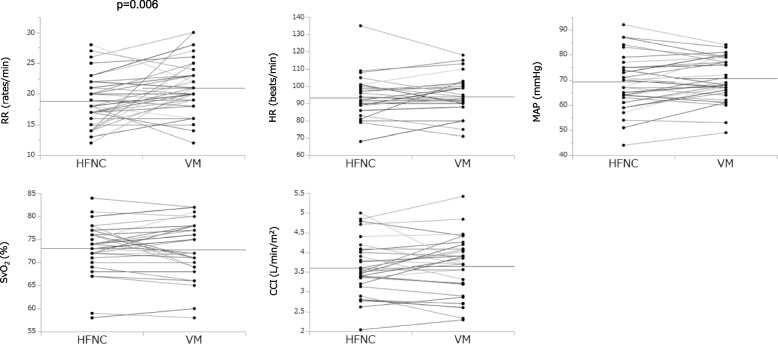
HFNC vs. VM (physiological data). HFNC, high flow nasal cannula; VM, venture mask; RR, respiratory rate; HR, heart rate; MAP, mean arterial pressure; CCI, continuous cardiac index; SvO_2_, mixed venous oxygen saturation. Horizontal lines indicate the mean value of each device

PaO_2_ was significantly higher in the HFNC group than in the VM group [101.7 (25.9) vs. 91.8 (23.0), mean difference 9.87 (18.5), 95% confidence interval 3.5 to 16.2, *p* = 0.003]. As well, PFR was significantly higher in the HFNC group than in the VM group [265.9 (81.4) vs. 238.7 (68.5), mean difference 27.2 (49.1), 95% confidence interval (10.3 to 44.1), *p* = 0.002]. Moreover, PaCO_2_ was slightly, but significantly, lower in the HFNC group than in the VM group [33.8 (3.5) vs. 34.7 (2.9), mean difference − 0.95 (2.5), 95% confidence interval (− 1.84 to − 0.06), *p* = 0.033] (Fig. 2). As for the physiological data, the respiratory rate was significantly lower in the HFNC group than in the VM group [18 (4) vs. 21 (4), mean difference − 2.2 (4.47), 95% confidence interval (− 3.74 to − 0.66), *p* = 0.006], and no significant differences were seen in any of the other parameters (Fig. 3). Tables 4, 5, 6 and 7 show a comparison of outcome variables separately performed for protocol A and protocol B. Similarly to the crossover analysis, PFR was significantly higher in HFNC for both protocols. PCO_2_ was significantly lower in HFNC for protocol A but not for protocol B. RR was significantly lower in HFNC for protocol B, but not for protocol A.

**Table 4:** Comparison of arterial blood gas analysis in protocol A

	VM		HFNC		mean diffference	95%CI			*p* value
PFR (mmHg)	243.7	(62.5)	272.0	(88.6)	−28.3	− 53.8	to	− 2.9	0.031
PO_2_ (mmHg)	90.7	(18.8)	101.0	(27.4)	−10.3	−19.7	to	−0.9	0.034
PCO_2_ (mmHg)	34.6	(3.0)	32.4	(3.6)	2.1	0.9	to	3.3	0.002
HCO_3_^−^ (mEq/L)	22.7	(2.3)	22.1	(2.6)	0.5	−0.2	to	1.2	0.129
pH	7.44	(0.03)	7.45	(0.03)	−0.01	−0.03	to	−0.00	0.003

*HFNC* High flow nasal cannula, *VM* Venture mask, *CI* Confidence interval, *PFR* PaO_2_/FiO_2_ ratio

Data are presented as mean (standard deviation)

**Table 5:** Comparison of physiological data in protocol A

	VM		HFNC		mean diffference	95%CI			*p* value
RR (rates/min)	20.5	(5.3)	18.7	(3.6)	1.8	−0.5	to	4.2	0.118
HR (beats/min)	91.2	(10.6)	93.6	(13.0)	−2.4	−6.6	to	1.9	0.262
MAP (mmHg)	69.1	(9.7)	69.4	(12.1)	−0.3	−2.9	to	2.3	0.811
SvO_2_ (%)	70.3	(12.4)	70.7	(12.4)	−0.4	−2.6	to	1.7	0.673
CCI (L/min/m^2^)	3.7	(0.9)	3.7	(0.7)	0.0	−0.2	to	0.2	0.970

*HFNC* High flow nasal cannula, *VM* Venture mask, *CI* Confidence interval, *RR* Respiratory rate, *HR* Heart rate, *MAP* Mean arterial pressure, *SvO*_*2*_ Mixed venous oxygen saturation, *CCI* Continuous cardiac index

Data are presented as mean (standard deviation)

**Table 6:** Comparison of arterial blood gas analysis in protocol B

	HFNC		VM		mean difference	95%CI			*p* value
PFR (mmHg)	260.2	(76.2)	234.0	(75.3)	26.2	1.3	to	51.1	0.041
PO_2_ (mmHg)	102.3	(25.1)	92.8	(26.9)	9.5	−0.1	to	19.1	0.052
PCO_2_ (mmHg)	35.0	(3.0)	34.9	(2.9)	0.1	−1.0	to	1.3	0.791
HCO_3_^−^ (mEq/L)	23.1	(2.0)	23.1	(1.5)	0.0	−0.7	to	0.7	0.935
pH	7.43	(0.04)	7.44	(0.03)	−0.01	−0.02	to	0.00	0.254

*HFNC* High flow nasal cannula, *VM* Venture mask, *CI* Confidence interval, *PFR* PaO_2_/FiO_2_ ratio

Data are presented as mean (standard deviation)

**Table 7:** Comparison of physiological data in protocol B

	HFNC		VM		mean difference	95%CI			*p* value
RR (rates/min)	18.9	(4.5)	21.4	(3.0)	−2.6	−4.8	to	−0.3	0.028
HR (beats/min)	92.9	(9.0)	96.3	(9.1)	−3.4	−5.4	to	−1.4	0.002
MAP (mmHg)	72.2	(6.8)	69.2	(9.3)	3.0	0.3	to	5.7	0.033
SvO_2_ (%)	73.2	(3.5)	72.9	(4.8)	0.3	−1.5	to	2.1	0.728
CCI (L/min/m^2^)	3.5	(0.7)	3.6	(0.6)	−0.1	−0.3	to	0.2	0.626

*HFNC* High flow nasal cannula, *VM* Venture mask, *CI* Confidence interval, *RR* Respiratory rate, *HR* Heart rate, *MAP* Mean arterial pressure, *SvO*_*2*_ Mixed venous oxygen saturation, *CCI* Continuous cardiac index

Data are presented as mean (standard deviation)

## Discussion

This is the first randomized crossover trial to compare the short-term efficacy of HFNC and VM for cardiovascular surgical patients. Our study revealed that, compared with VM, HFNC ameliorates gas exchange, and that the hemodynamic state did not differ between these devices in cardiovascular surgical patients after extubation. In addition, using HFNC reduced the respiratory rate when the patient was switched from VM. These findings do not contradict a previous report that HFNC generates a flow-dependent effect of continuous positive airway pressure [2] and an upper airways deadspace washout effect [3, 4]. In addition, delivering humidified and heated oxygen reduces patient effort and oxygen consumption. The most distinctive characteristic of this study is that we directly measured the flow rate of VM by using the HALOSCALE® flowmeter when comparing the rate with HFNC. As for those studies [5, 6] using HFNC compared with VM, the method application of VM was not mentioned in detail. VM cannot provide pre-set oxygen concentration with inappropriate total flow rate of < 30 L/min.

In recent years, HFNC has been widely and rapidly propagated as a standard oxygen delivery system, especially for those patients with deteriorated oxygenation function. The results of recent randomized control trials show that HFNC, at a minimum, is not inferior to noninvasive ventilation (NIV) [7, 8]. Especially with regard to its comfortability, HFNC was thought to be superior to NIV. However, Elie Azoulay et al. demonstrated that HFNC therapy did not significantly decrease mortality among critically ill patients with acute respiratory failure, compared with standard oxygen therapy [9]. The greatest advantage of HFNC is its capability of adjusting both oxygen concentration (0.21 to 1.0) and total gas flow (0 to 60 L/min). Using a high flow rate of over 30–40 L/min, HFNC can provide a gas flow rate without decreasing oxygen concentration due to air entrainment. VM is also capable of adjusting both oxygen concentration and total gas flow rate; however, its adjustable range is restricted (Table.1). Although several randomized control trials for cardiovascular surgical patients were carried out comparing HFNC with conventional oxygen therapy such as VM or face mask with a reservoir bag, HFNC ameliorated oxygenation but did not decrease perioperative mortality [5, 10]. On the other hand, focusing on short-term therapeutic effects, various verifications have been made. After cardiothoracic surgery, a postoperative routine use of HFNC did not yield improvement in oxygenation nor reduce the rate of atelectasis; however, it did reduce the requirement for an escalation of respiratory support, such as a high flow face mask, HFNC, NIV, and reintubation [11]. Maggiore et al. demonstrated that HFNC could provide an improvement in oxygenation only after 24 h of treatment for hypoxemic patients in their weaning from mechanical ventilation after acute respiratory failure [5]. A recent study compared the preemptive use of HFNC and VM after thoracotomy for lung resection. In the study, HFNC did not reduce the incidence of postoperative hypoxemia but did reduce the incidence of postoperative hypercapnia, compared to VM [12]. Although these findings suggest that HFNC does not ameliorate the long-term prognosis, it was beneficial for the postoperative patient to avoid hypoxemia or hypercapnia after extubation.

On the other hand, taking advantage of its excellent oxygenation, the validity of HFNC as a preoxygenation device has been reported [13]. Recently, the indication of HFNC usage has been developing, not only for the treatment of respiratory failure after extubation in the ICU, but also for preoxygenation before intubation in the emergent or operating room.

This study has some limitations, however. In it, we provided a 60-minute wash-out period after each device usage in order to eliminate the effects of the prior oxygen delivery. A previous study demonstrated that, after either an increase or decrease in FiO_2_ in stable condition, 5 to 10 minutes is adequate to accurately measure arterial blood samples [14]. Hence, 60 minutes of wash-out period is considered to be sufficient. During both protocols, FiO_2_ and total gas flow rate were maintained fixed and similar; however, FiO_2_ was not similar, actually, between these devices because the entrainment of room air varied during breathing when VM was used. In addition, the entrainment of room air with HFNC at 40 L/min could be substantially lower, as the peak inspiratory flow in stable patients after extubation should not exceed 40 L/min and, in this study, was lower than the HFNC setting. Therefore, the higher actual FiO_2_ could explain the higher PaO_2_ (Fig. 2) when using HFNC.

## Conclusion

Compared to VM, HFNC ameliorates oxygenation function and gas exchange and decreased patients’ effort in breathing. The hemodynamic state did not differ between HFNC and VM and, therefore, HFNC can be used safely in cardiovascular surgical patients after extubation.

## Data Availability

The datasets and analyses of this study are available from the corresponding author upon reasonable request.

## Abbreviations

ICU: Intensive care unit
HFNC: High flow nasal canulla
VM: Venturi mask
PFR: PaO_2_/FiO_2_ ratio
ABG: Arterial blood gas
RR: Respiratory rate
HR: Heart rate
MAP: Mean arterial pressure
CCI: Continuous cardiac index
SvO_2_: Mixed venous oxygen saturation
NIV: Noninvasive ventilation
